# Crying in the algorithm: modeling academic stress via multilayer topic construction and ERA effect

**DOI:** 10.3389/fpsyg.2025.1673559

**Published:** 2025-09-19

**Authors:** Liwei Ding, Hongfeng Zhang, Jinqiao Zhou

**Affiliations:** Faculty of Humanities and Social Sciences, Macao Polytechnic University, Macao, China

**Keywords:** academic pressure, multi level topic modeling, multilevel stress theory, sentiment analysis, expectancy-regulation-amplification (ERA) model

## Abstract

Amid intensifying educational competition and societal expectations, academic stress has emerged as a multidimensional force influencing student mental health. While prior research has explored individual and institutional factors, limited attention has been paid to how learners semantically construct and express academic stress in digital environments. Addressing this gap, this study introduces an innovative multilayered topic modeling framework that integrates BERTopic and Latent Dirichlet Allocation (LDA), enabling a semantic, data-driven analysis of 33,827 user-generated comments related to academic pressure on social media. Grounded in Multilevel Stress Theory, the analysis identifies six interrelated topics reflecting the interplay of individual, situational, and structural stressors. Drawing on these findings, the study develops the Expectancy–Regulation–Amplification (ERA) Model, which conceptualizes academic stress as a dynamic process shaped by the tension between external expectations and perceived capabilities, limitations in self-regulatory resources, and the cumulative amplification of stress across sociocultural and digital environments. By mapping how academic pressure is linguistically reproduced and sentimentally intensified in algorithmic settings, the ERA model provides an interpretive framework for understanding the semantics of student vulnerability and contributes new insights to targeted interventions in educational and mental health contexts.

## 1 Introduction

With the rise of international competition in education and rising educational achievements, academic pressure is today among the most pressing challenges that are demoralizing the psychological well-being of students (Clabaugh et al., 2021). Extensive research has proved that excessive academic pressure not only threatens the psychological well-being of students but can even cause long-term negative effects on their study performances (Steare et al., 2023; Wuthrich et al., 2020). This is no longer merely the product of individual scholarship burdens alone; rather, it is formed by the reciprocating play of several interacting forces that encompass the education system, parental expectation, and societal judgments, gradually emerging as a structural and institutional social phenomenon (Grossi et al., 2020; Pajarianto, 2020). In particular, within the scenario of standardized testing, scoring assessments, and selection through elite procedures—the system for curriculum evaluation well established—students’ burden has long surpassed the fear of “not learning.” It has instead become more severe physical and psychological afflictions, proceeding towards existential disorientation (Grossi et al., 2020; Ye et al., 2025).

Academic stress is not only an outcome of the learning process but has also emerged as a very powerful motivating factor for the rise in mental health issues during the past decade (Keyes and Platt, 2024). Initial studies predominantly focused on the correlation between academic stress and mental health among adolescents through clinical observations (Altmann and Gotlib, 1988) and qualitative interviews (Andrews et al., 2006). Quantitative approaches have come to dominate over time, though arising from the interdisiplinary crossroads of psychology, pedagogy, and sociology (Reddy et al., 2018). The COVID-19 global crisis precipitated the mass adoption of online learning, and researchers were consequently encouraged to investigate the potential impact of digital uncertainty on learning motivation as well as psychological stress (Moawad, 2020; Ye et al., 2020). Further, the advent of artificial intelligence and big data technologies has taken stress research to unprecedented levels, enabling extraction of semantic patterns from unstructured data, such as social media, and large-scale, multi-level quantification and tracking of stress (Ali et al., 2024; Ratul et al., 2023).

The existing literature has completely illuminated the long-term effects of academic stress on motivation to learn, psychological distress, and self-efficacy. For instance, earlier work has placed more importance on the predictive nature of stress concerning mental health issues (Córdova Olivera et al., 2023), such that sentimental disturbance has a tendency to evoke a vicious circle involving stress, worry, and school failure (Kristensen et al., 2023). Other research establishes the multifaceted association between self-determined motivation and scholarly stress, with findings that scholarly stress, namely, grades-related anxiety, may decrease intrinsic motivation. Such reduction might be experienced within a few months, which can develop into depression in the long term (Yang et al., 2022). Beyond the psychological dimensions, some studies suggest that external social factors exert a more pronounced influence (Deng et al., 2022; Wahid et al., 2023). For example, in research conducted within Islamic schools, a significant correlation was found between academic stress and parenting styles, with democratic parenting particularly influencing stress levels (Dwi Utari and Hamid, 2021). Similarly, in South Korea, high school students are grappling with escalating academic pressures, driven by fierce competition, societal expectations, social media, and peer influence. These pressures are compounded by the absence of adequate psychological support systems (Ji-hoon, 2024).

Despite a wealth of studies investigating the factors that contribute to academic stress, there remains a notable absence of systematic exploration and semantic modeling of how this stress is articulated through language and psychologically expressed on community platforms. In particular, how stress is “voiced” through language and subsequently replicated in platform interactions remains only partially known. In order to close this evidence gap, the present paper takes the Multilevel Stress Theory as its central analytical tool. Based on this theory, it is argued that stress arises from the interaction of individual, situational, and structural causes (Probst, 2010; Stranks, 2005). The anxiety experienced by individuals—whether stemming from exam tension, the uncertainty of academic outcomes, or feelings of diminished self-worth—often intersects with external environmental pressures such as familial expectations, societal evaluation, and institutional structures, thereby intensifying the perception of stress. This confluence of multiple factors fosters both cumulative stress (Morales and Guerra, 2006) and an interactive amplification of pressure (Hallett, 2003). This three-dimensional framework offers a more nuanced understanding of the social mechanisms that underpin individual sentimental experiences.

From a methodological perspective, this study combines BERT-based Topic Modeling (BERTopic) with the Latent Dirichlet Allocation (LDA) model to conduct multilevel topic modeling and semantic chain analysis of user comments related to academic stress on social media platforms like YouTube. This approach aims to extract core meaning configurations and underlying cultural-psychological tensions across topics. However, since the data are sourced from YouTube comments within a specific time frame and region, the contextual background is difficult to pinpoint accurately. Additionally, data collection across different countries and time points may not fully capture cultural and societal differences. Future research should expand the data collection scope to more accurately capture the impact of different contexts on academic stress and to validate the generalizability of the theoretical framework.

Using language as an entry point, this study seeks to reveal how experiences of stress emerge within digital narratives and social media expressions, thereby responding to recent research trends on how language reflects and reconstructs psychological states. In summary, the study aims to investigate the structural generative logic of academic stress and its linguistic constructional features, as well as to analyze how individuals express and respond to stress under the multiple disciplines of belief, education, and digital environments. In doing so, this research not only redefines academic stress at the semantic level—focusing on sentiments rather than sentiments—but also provides multidimensional perspectives and theoretical foundations for educational interventions and psychological support strategies.

## 2 Research design and methodology

**Figure 1:**
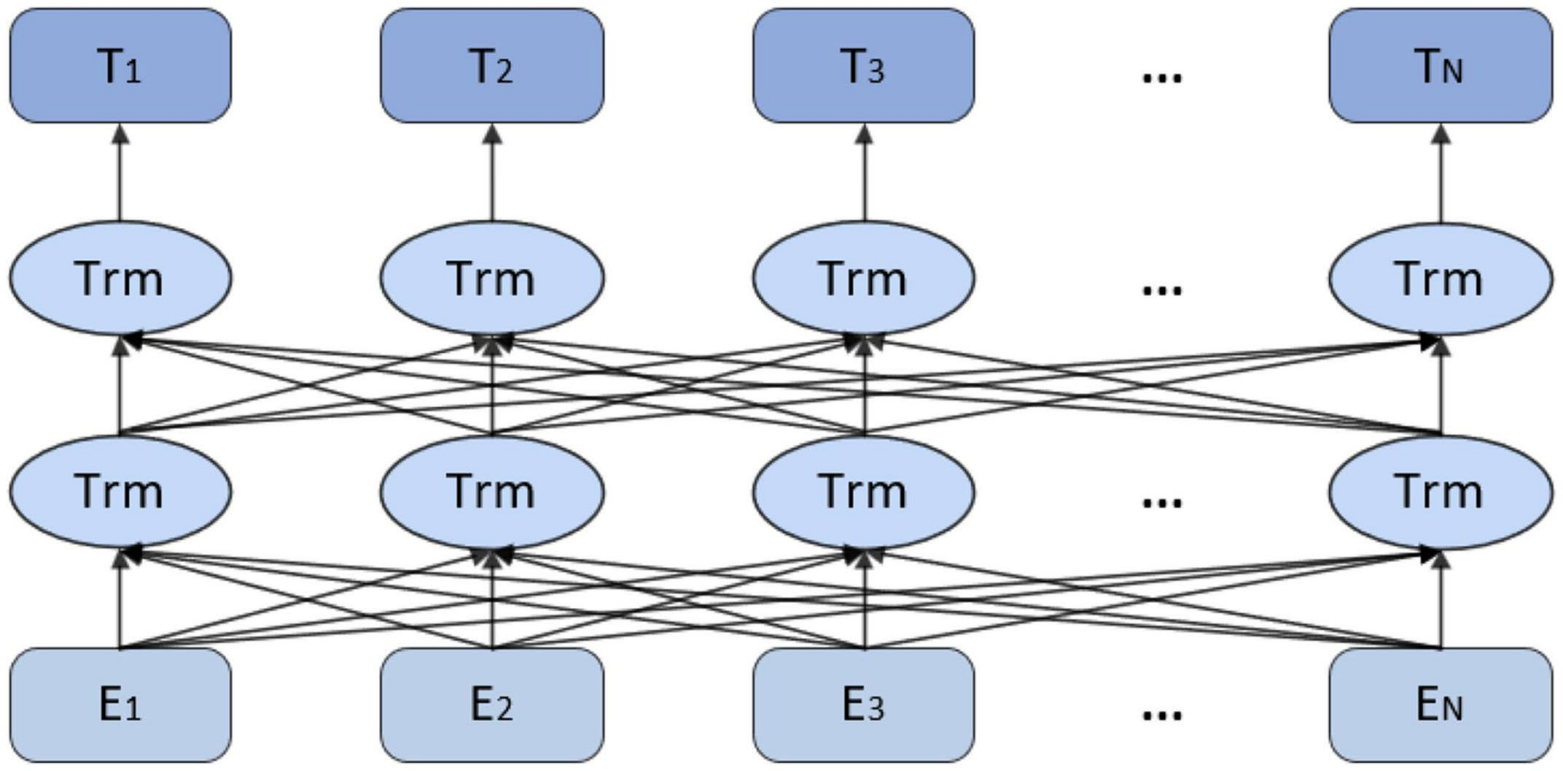
Thought diagram of BERT’s built-in neural network.

This study adopts a hybrid methodology, combining multi level topic modelling and sentiment analysis. By the integration of theme extraction and sentiment detection, we delve deep into the multi-dimensional aspects of information embedded in the text. The primary purpose of this research is to probe the underlying mechanisms of academic stress and to examine how various determinants blend with the coping strategy of students. BERT (Bidirectional Encoder Representations from Transformers) is a pre-trained transformer language model that has gained popularity due to its subtlety in text analysis via bidirectional contextual understanding (Koroteev, 2021). BERT sentiment analysis is a text classification method based on a pre-trained language model, which determines the sentiment orientation of an input text by modeling its contextual semantics. Unlike traditional unidirectional language models, BERT simultaneously considers both the preceding and following context during text processing. This bidirectional understanding enables more accurate sentiment classification, improving both the precision and granularity of sentiment analysis (Bouraoui et al., 2020). Figure 1 illustrates the conceptual flow of BERT’s internal neural network processing.

Currently, BERTopic has emerged as an innovative topic modeling technique in the field of text mining. As a deep learning–based method, BERTopic employs the BERT model to generate text embeddings, capturing semantic relations within texts, and applies clustering algorithms to extract semantically rich topics (Bouraoui et al., 2020). BERTopic is built upon the Transformer framework to generate context-sensitive vector representations for each text. Typically, this is calculated using the following equation (1), where hi represents the embedding of the text xi, and BERT(xi) denotes the context-sensitive embedding vector of the text xi produced by the BERT model. Through this process, BERTopic is able to capture not only semantic structures but also underlying sentiments and sentiment-related variations embedded within texts, thereby providing deeper insights into both thematic and affective dimensions of discourse.


(1) $$h_{i}=\text{BERT}(x_{i})$$

While the BERTopic method excels at uncovering potential topic structures within text through semantic-based extraction, and is effective in identifying semantic relationships and topic associations within a document, it is not without its limitations. In certain instances, BERTopic struggles to differentiate between vocabulary that shares similar semantic expressions. Furthermore, clustering algorithms often face challenges when confronted with semantic diversity and domain-specific variations, leading to the misclassification of unrelated vocabulary under the same topic. Both BERTopic and LDA offer distinct advantages (Egger and Yu, 2022). To address this issue, LDA is introduced as a complementary approach, effectively compensating for the shortcomings of BERTopic. As opposed to BERTopic, which depends so heavily on text embedding, LDA pays more attention to word distribution and co-occurrence, thereby enhancing and refining semantic segregation of topics.

LDA is a statistical topic modeling technique employed in identifying hidden thematic structure in a set of documents (Jelodar et al., 2019). LDA predicts that documents are generated from a mixture of topics and that each topic possesses some probability distribution over a word vocabulary. The core process in LDA is either Gibbs sampling or variational inference, which are applied to estimate parameters from data and subsequently infer document-topic distribution and word-topic distribution for every topic (Yu and Xiang, 2023). With the use of BERTopic, LDA also helps in surmounting the difficulty in recognizing semantically similar expressions posed by the latter by leveraging its probabilistic model. This enhancement enables better topic identification of documents. The expression in equation (2) describes the topic assignment mechanism in the document generation model of the LDA model. Given the topic distribution *θ*_d_ of a document, we sample a topic Z_i_ from it to assign a topic to the i-th word in the document.


(2) $$Z_{i}\sim \text{Multinomial}(\theta_{d})$$

**Figure 2:**
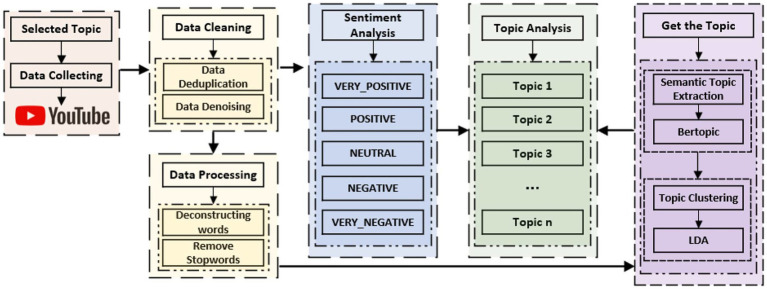
General diagram of research methods.

Throughout this study, considerable amounts of review data were initially gathered, thereafter cleaned and prepared extensively. Subsequent to this, the sentimental bias of the content was analyzed through the use of sentiment detection technology. This was thereafter merged with both the BERTopic and LDA models to establish the underlying topics of the data. Finally, there was a close-up examination of the inner organization and affective relationships of each topic to reveal the underlying sentimental and thematic patterns in the dataset. As Figure 2 illustrates, the most critical research steps in the process, including data gathering, sentiment recognition, and topic identification, are outlined.

## 3 Methodology

### 3.1 Platform selection

In the preliminary phase of this study, we selected several social media platforms for data collection, including TikTok, X (formerly Twitter), and YouTube—platforms with high user activity levels. However, during the data collection process, we compared and analyzed the content quality across these platforms and found that discussions on TikTok and X were inconsistent, with a significant amount of advertising and irrelevant content, severely affecting the data’s validity. After further searching, the remaining valid data from TikTok and X was limited and did not meet the expected quality standards. In contrast, YouTube presented data of significantly higher quality than the other two platforms. Although some advertisements were present, most of the content was highly relevant and valuable for research, particularly with a wealth of genuine user experiences and personalized expressions concerning academic stress. After a rigorous filtering and exclusion process, this study retained 33,827 valid entries out of approximately 60,000 raw data collected from YouTube, resulting in a data utilization rate of about 55%. Based on the analysis and quality considerations outlined above, YouTube was ultimately established as the primary data source to ensure the scientific accuracy and reliability of the research findings. This study chose YouTube as the source of data, which is among the globe’s largest and most populous video-sharing websites (Alhujaili and Yafooz, 2021). Furthermore, the website not only contains individual student accounts and peer talks about stress reactions, but also professional guidance, such as mental health guidance and stress coping mechanisms from psychologists and educators. Such diverse contents provide an efficient means of examining the psychological states and adaptation modes of contemporary students faced with scholarly pressure, psychological contradiction, and crises of belief. They provide valuable data and empirical facts for research in related fields, so that the authenticity and practicability of the results obtained in this study will be enhanced. For instance, Table 1 indicates five remarks obtained from YouTube.

**Table 1:** Comment data table.

Number	Related topic	Comments	Sentiment labels
1	Topic 2, Topic 4, Topic 6	Recently I’ve been driven to burnout, anxiety, and depression (mainly cause of some family life things). Even though I’m not studying in the traditional sense, this video was exactly what I needed.	VERY_POSITIVE
2	Topic 1, Topic 6	Well, he certainly summed up Existential nihilism. A person can find a subjective meaning to their life but there is simply is no purpose to any of our lives.	POSITIVE
3	Topic 3, Topic 6	We can make a better world through Christianity and being able to embrace our imperfections. One person can make a change and that change can be you.	NEUTRAL
4	Topic 1, Topic 2, Topic 5, Topic 6	I got − 5 in my exam and teacher complained about me.	NEGATIVE
5	Topic 2, Topic 4	Depression is the label given by a social and economic system, to people who dislike what life actually.	VERY_NEGATIVE

### 3.2 Data acquisition and preprocessing

This study, in strict adherence to YouTube’s Terms of Service and Data Usage Policy, utilized the official YouTube Data API interface to collect data from publicly available pages on the platform. The study focused on the keyword “academic stress” to perform data scraping and precise searching on YouTube. It selected videos with significant social impact from the past 5 years and systematically collected their public comments. The choice of “academic stress” as the primary keyword encompasses both objective descriptions of stress and sentimental expressions, ensuring the comprehensiveness of the corpus. In order to confirm the appropriateness of the keywords and the credibility of the study, we also tested other related phrases (such as “exam stress,” “academic anxiety,” “student learning psychology” etc.) during the initial research process. Keywords are closely associated with the research topic and best reflect students’ psychological and sentimental experiences within academic pressures. Ultimately, the study collected over 80,000 valid comments, providing a good source of primary data for the analysis that followed.

The analysis phase of the current study was carried out using PyCharm, a robust integrated development environment specifically made for Python programming. Following data collection, the raw corpus passed through systematic preprocessing involving cleaning, filtering, building a preprocessing dictionary, tokenization, and stop word removal. The research utilized the langdetect library (a Python library used to automatically identify the language of the input text) to confirm that the corpus was in English from start to finish. Then, automated procedures were applied to deduplicate and strip emojis. Furthermore, both manual and machine-aided verification was employed in order to filter out noise data such as spam and profanity. To this end, a preprocessing dictionary was built step by step, which normalized spelling, slang, and colloquial expressions, as well as tagging sentiment and topics to improve the accuracy of subsequent analysis. This pre-processing lexicon included empty high-frequency words and phrases, emoji to word conversions, and served both data denoising and structural processing. It also helped in correct identification of certain semantic fields such as “academic stress” and “academic anxiety.” Tokenization and stop word removal were subsequently performed through the Natural Language Toolkit (NLTK), a widely used Python library designed to provide comprehensive support for natural language processing. With these procedures, the data was once more deduplicated. By running several cycles of normalization and cleaning, 33,827 clean comments were left and thus a high-quality and clean corpus was formed for future research.

### 3.3 Data in-depth analysis

#### 3.3.1 Sentiment analysis

To understand the specific sentiment tendencies of the public under academic pressure, this study employs BERT for sentiment analysis (Bello et al., 2023). The model captures more complex semantic relationships in text, and with the Transformers library’s convenience in processing natural language tasks, it enables efficient sentiment analysis of large volumes of comment data. After cleaning and preprocessing the data, the sentiment analysis loaded the BERT-based model nlptown/bert-base-multilingual-uncased-sentiment (a pre-trained BERT model already trained on sentiment analysis tasks) to perform the sentiment analysis task. This model is a five-class sentiment analysis model, with sentiment labels corresponding to five sentiment tendencies: VERY_POSITIVE, POSITIVE, NEUTRAL, NEUTRAL, and VERY_NEGATIVE. VERY_POSITIVE and POSITIVE represent high-intensity and low-intensity positive sentiments, respectively; NEUTRAL indicates neutral sentiment with no significant intensity; while NEGATIVE and VERY_NEGATIVE represent low-intensity and high-intensity negative sentiments, respectively. Table 1 displays the five training dataset information along with the results after BERT sentiment analysis. Compared to traditional binary classification (positive/negative) or three-class classification (positive/neutral/negative) methods, this detailed classification system can more precisely capture the differences in sentiment tendencies within the comments, thereby reflecting the hierarchical and complex nature of the comment data more comprehensively. For example, under the specific theme of academic pressure, the public’s sentiments may not be simply positive or negative, but rather exhibit varying levels of sentimental intensity. Therefore, this multi-level labeling system can provide richer information, helping the study to gain a deeper understanding of the public’s sentimental fluctuations.

To systematically evaluate the performance of the BERT model in sentiment analysis tasks, this study employed a random sampling method for testing. In each instance, 200 samples were randomly selected from the original corpus, and five independent samplings were conducted, ultimately forming a test set of 1,000 data points. The testing process in this study adopted a “random selection + expert evaluation of sentiment labels” approach, where domain experts manually annotated each sample to create the ground truth labels, while the sentiment analysis performed by the BERT-based five-class model produced predicted labels. Based on this, the study calculated the model’s Accuracy, Precision, and F1 Score, with the results for each metric presented in Table 2. From the results of the five tests, the BERT model demonstrated good performance in both Precision and F1 Score, indicating strong performance in the task of identifying positive sentiment, and effectively distinguishing between positive and negative sentiments. However, the fluctuations in experimental results suggest the existence of potential uncertainties. These differences partly stem from the inherent subjectivity in the manual annotation process and the variability of annotation standards, meaning that the ground truth labels could still contain some bias. Furthermore, the randomness in sample extraction and the representativeness of the test set may also impact the results, leading to differences in performance across different batches.

**Table 2:** BERT sentiment analysis test parameters:

Group	Accuracy	Precision	F1 Score
1	0.79	0.89	0.82
2	0.84	0.92	0.85
3	0.89	0.92	0.90
4	0.84	0.92	0.85
5	0.79	0.89	0.82

#### 3.3.2 Obtaining topics

This study used the BERTopic method to perform topic modeling analysis on 33,827 text data points. First, the SentenceTransformer model was used to convert the text data into embedding vectors, which effectively capture the semantic information of the text. Next, the UMAP dimensionality reduction algorithm was applied to map the high-dimensional embedding vectors to a lower dimension, thereby improving the efficiency of clustering. Then, the HDBSCAN clustering algorithm was used to cluster the reduced-dimensional embedding vectors, grouping semantically similar texts into the same topic. Additionally, to further enrich the understanding of the topics, this study employed phrase mining techniques to extract core vocabulary from the texts, which helped better reveal the core content of each topic. Through this process, each topic was associated with 10 keywords, and the model ultimately identified 4,220 core terms (listed in rows corresponding to each topic in the output Excel table). Based on the semantic features and implications of these keywords, 422 topics were identified. For example, the terms depress, anxiety, study, exam, homework, pressure, time, review, illness, tried were linked to a topic related to study pressure and mental health. The semantic features and implications of this topic suggest that study pressure is not only a result of academic burdens but also influenced by sentiments, health, and psychological states across multiple dimensions.

**Figure 3:**
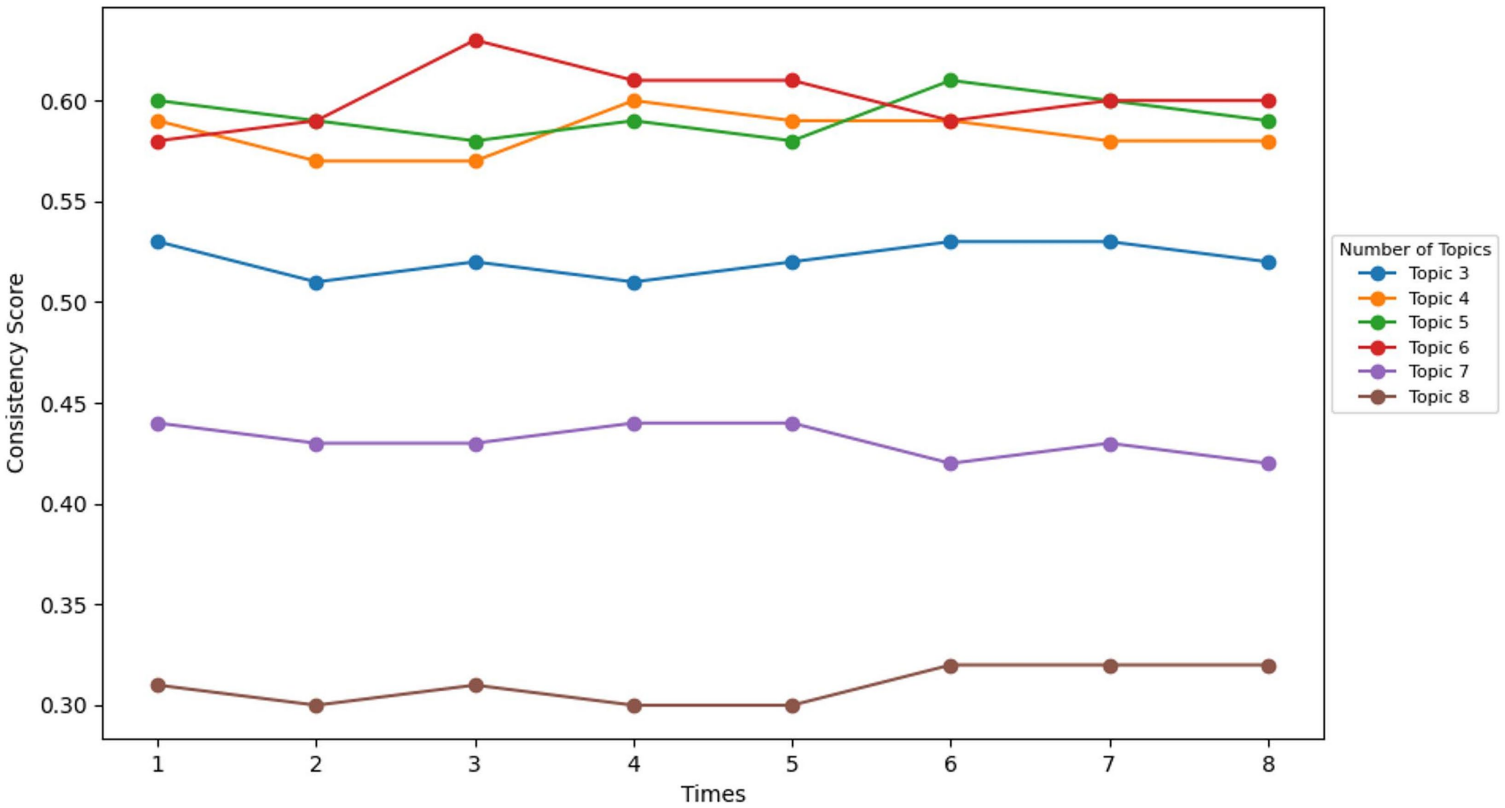
General diagram of research methods.

In this study, preliminary topic mining analysis revealed 422 potential topics, but not all of these topics showed a significant correlation with academic pressure. Therefore, to further identify topics that are significantly related to academic pressure, this study applied the LDA model. Keywords corresponding to each topic were generated into an Excel sheet to serve as the data source for the next round of analysis. Through the second clustering using LDA topic modeling, the study effectively extracted representative topics and core vocabulary. Compared to BERTopic, LDA was able to more clearly identify the relationships between topics. To ensure that the topics extracted through LDA modeling had high quality and representativeness, the study used the CoherenceModel class from the Gensim library (an open-source Python library commonly used for topic modeling and document similarity analysis) to calculate the coherence score of the topic model. The c_v scoring method was used here, which evaluates topic coherence by calculating word co-occurrence relationships and differences. As shown in Figure 3, the test results for the number of topics ranging from 3 to 8 indicated that the optimal number of topics was 6, with the coherence score fluctuating around 0.60, reflecting good topic coherence. Therefore, this was determined to be the optimal number of topics for LDA topic modeling.

## 4 Results

### 4.1 Sentimental attitude

**Figure 4:**
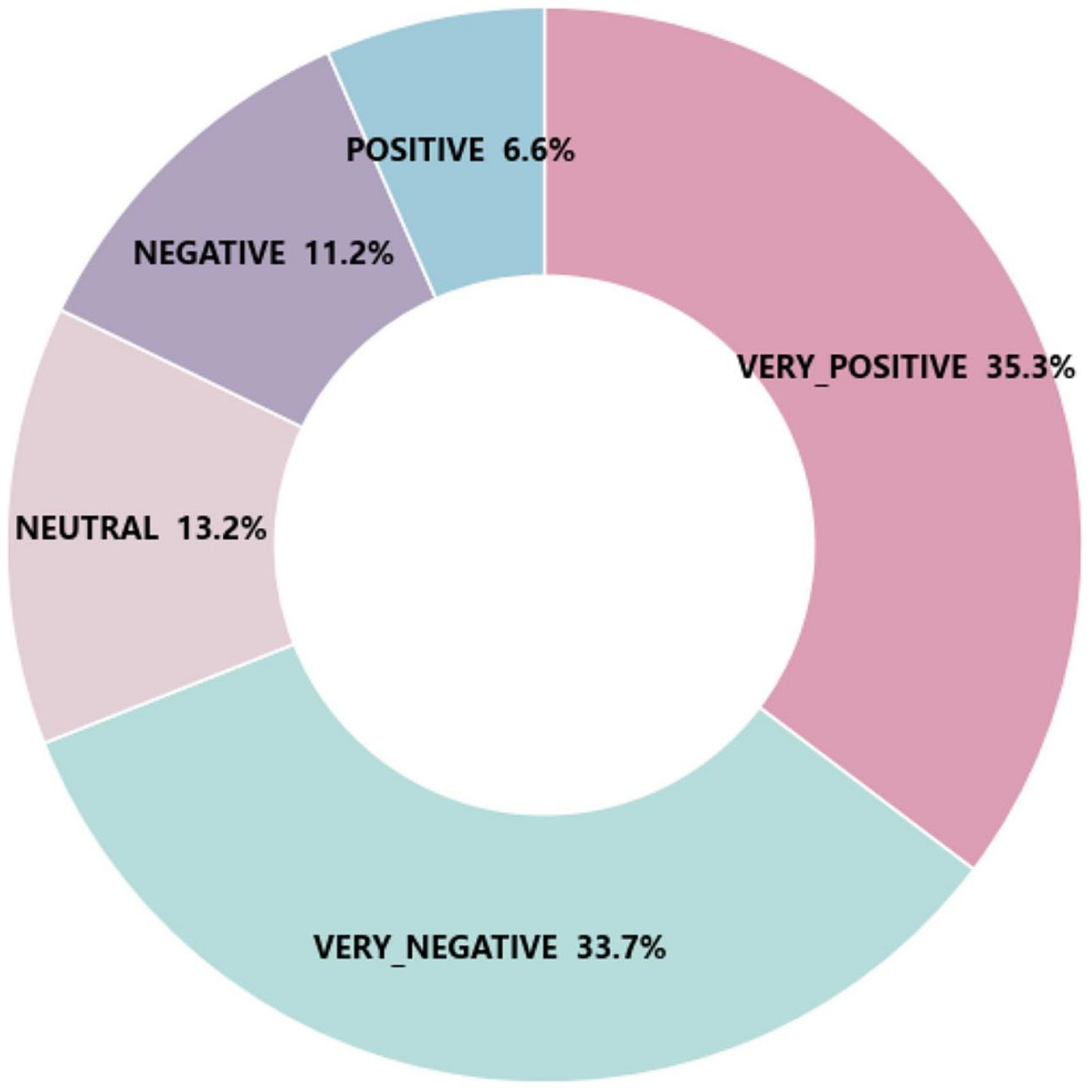
Sentiment percentage distribution chart.

After conducting sentiment classification on 33,827 comments using the BERT model, the results (as shown in Figure 4) reveal a complex distribution of sentiments. Specifically, the proportions of VERY_POSITIVE and POSITIVE are 35.3 and 6.6%, respectively, indicating that despite the presence of academic pressure, some students are still able to maintain positive sentiment orientations. In terms of sentiment intensity, VERY_POSITIVE represents highly intense positive sentiments, suggesting that these students demonstrate strong optimism and proactive coping mechanisms under stressful circumstances. In contrast, POSITIVE reflects milder positive sentiments, still indicating a positive sentiment orientation but at a lower intensity. The proportion of NEUTRAL stands at 13.2%, showing that some students exhibit a relatively stable sentimental state when facing academic pressure, without a pronounced sentiment orientation. Simultaneously, the relatively high rates of NEGATIVE (11.2%) and VERY_NEGATIVE (33.7%) indicate a clear distribution of negative feelings. NEGATIVE describes weak negative sentiments. Representing the reality that although some students experience negative affect, the degree of disturbance is moderate. In contrast, VERY_NEGATIVE illustrates high-grade sentimental distress, with the suggestion that most students live through tremendous extremes of negative sentiments under study pressure, perhaps in combination with psychiatric disorders such as depression or anxiety. Table 1 presents the distribution of comments across the five sentiment categories. This sentiment distribution pattern illustrates that students’ affective responses to academic pressure are not unidimensional but rather multi-layered and multidimensional. The combination of sentiment orientation and sentiment intensity provides a more nuanced and richer analysis of affective responses.

This result implies that university pressure has a significant effect on students’ feelings in today’s learning environment. As much as some students will learn adaptive coping skills, the majority of them grapple with negative sentiments such as anxiety, stress, and disappointment (Corwin et al., 2022). This multi-level dispensation of sentiments illustrates the non-linear nature of the impact of scholarly pressure on the sentimental response of students, implying that sentimental responses vary significantly from individual to individual and are derived through the complex interaction of many factors.

### 4.2 BERTopic construction

**Figure 5:**
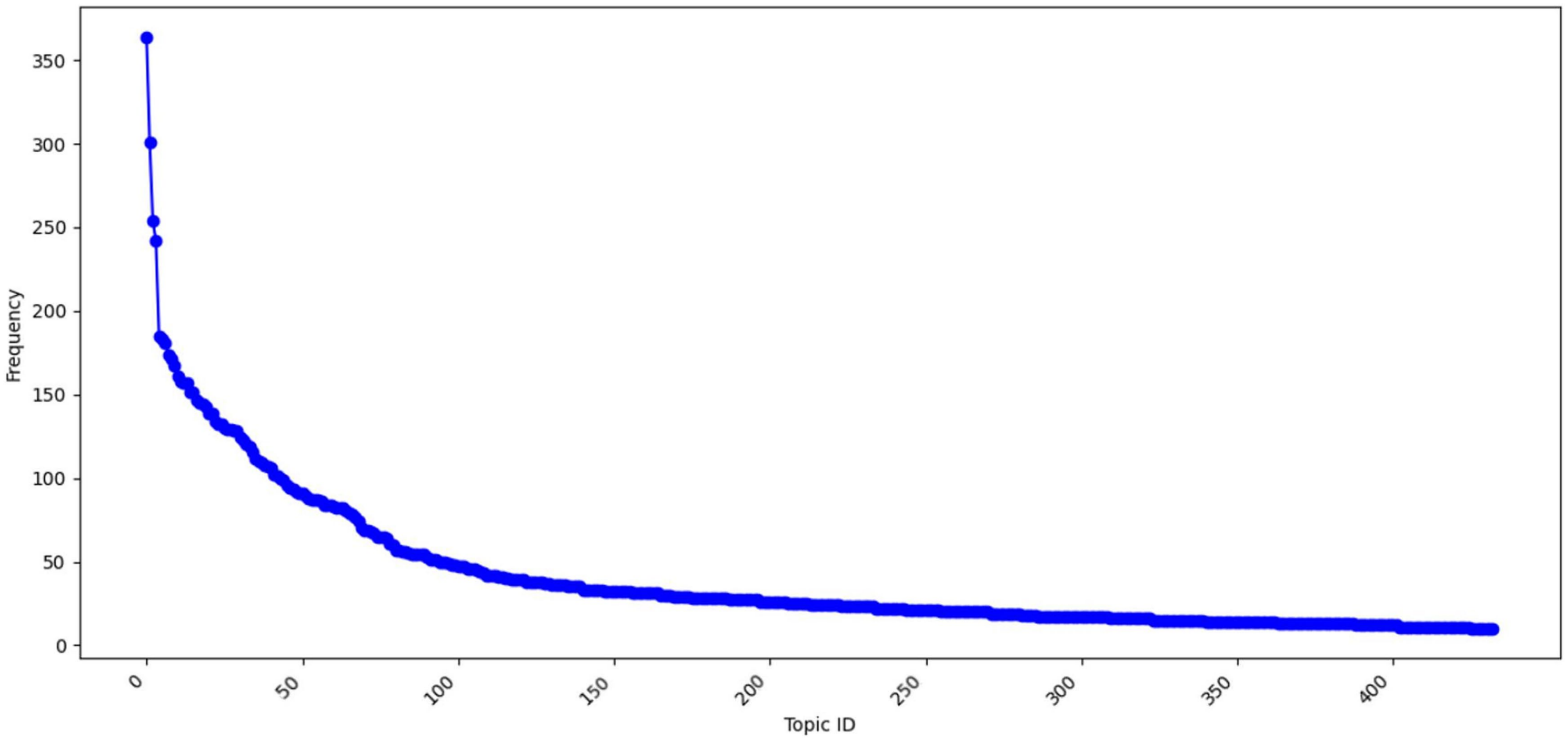
BERTopic all topics are displayed.

**Figure 6:**
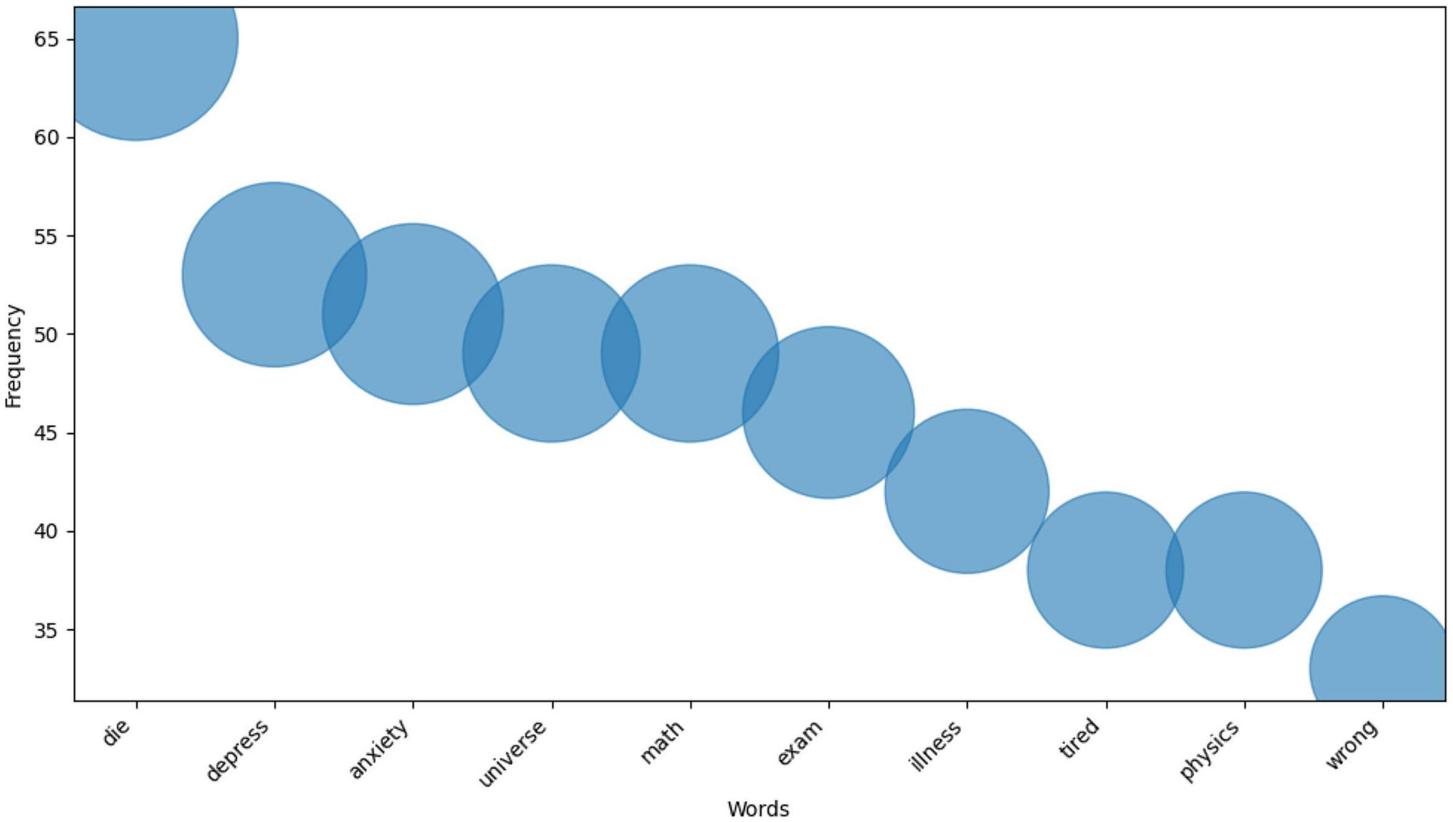
Top 10 topic words of Bertopic.

For conducting a more detailed study of academic stress, the betopic model was utilized for analyzing the comment data. Figure 5 shows the 422 topics generated by BERTopic and the frequency distribution of the respective comment data. It is noted that the four topics from Topic 0 to Topic 3 are connected with over 230 comments, among which the most significant one is Topic 0, which has over 350 comments. Figure 6 indicates the top 10 frequent words obtained from the BERTopic model and their occurrences. Based on these terms, it is clear that the comment content primarily revolves around academic pressure and sentiments of low sentimental tendencies, reflecting terminology and sentimental expressions related to subject exams. This reveals the core issues that commenters focus on, such as academic performance, environmental pressure, and psychological state.

Each topic contains 10 key vocabulary terms. In Topic 0, the vocabulary predominantly centres on terms that convey feelings of depression, such as “depression,” “anxiety,” and “tired.” In contrast, Topic 4’s vocabulary is largely focused on natural science-related terms, such as “universe,” “physics,” “atom,” and “galaxies,” reflecting a relatively lower match with the primary research topics. Statistical analysis of these topic vocabularies, as shown in Figure 6, highlights that terms like “universe” and “physics” appear among the top 10. For instance, one comment reads, “Stop arguing people, there is no gawd, and the universe does not give a shit about you so just live your life, do good and be good.” Here, “universe” serves more as a philosophical or sentimental symbol, reflecting concerns about the “God of Life” and encouraging individuals to focus on their own lives (Bennett et al., 2022). Conversely, another comment states, “Bio and chem are both crazy hard subjects. Oh and physics too.” In this case, “physics” refers directly to the academic subject, expressing the view that it is particularly challenging and difficult. Although betopic demonstrates certain strengths in topic modelling and vocabulary extraction, it does encounter limitations when dealing with terms that possess multiple contexts and connotations. Therefore, further optimisation of the model is required to enhance its accuracy and analytical capabilities within a multidimensional context, enabling it to more effectively capture the varied semantics of vocabulary.

### 4.3 LDA theme semantic analysis

**Figure 7:**
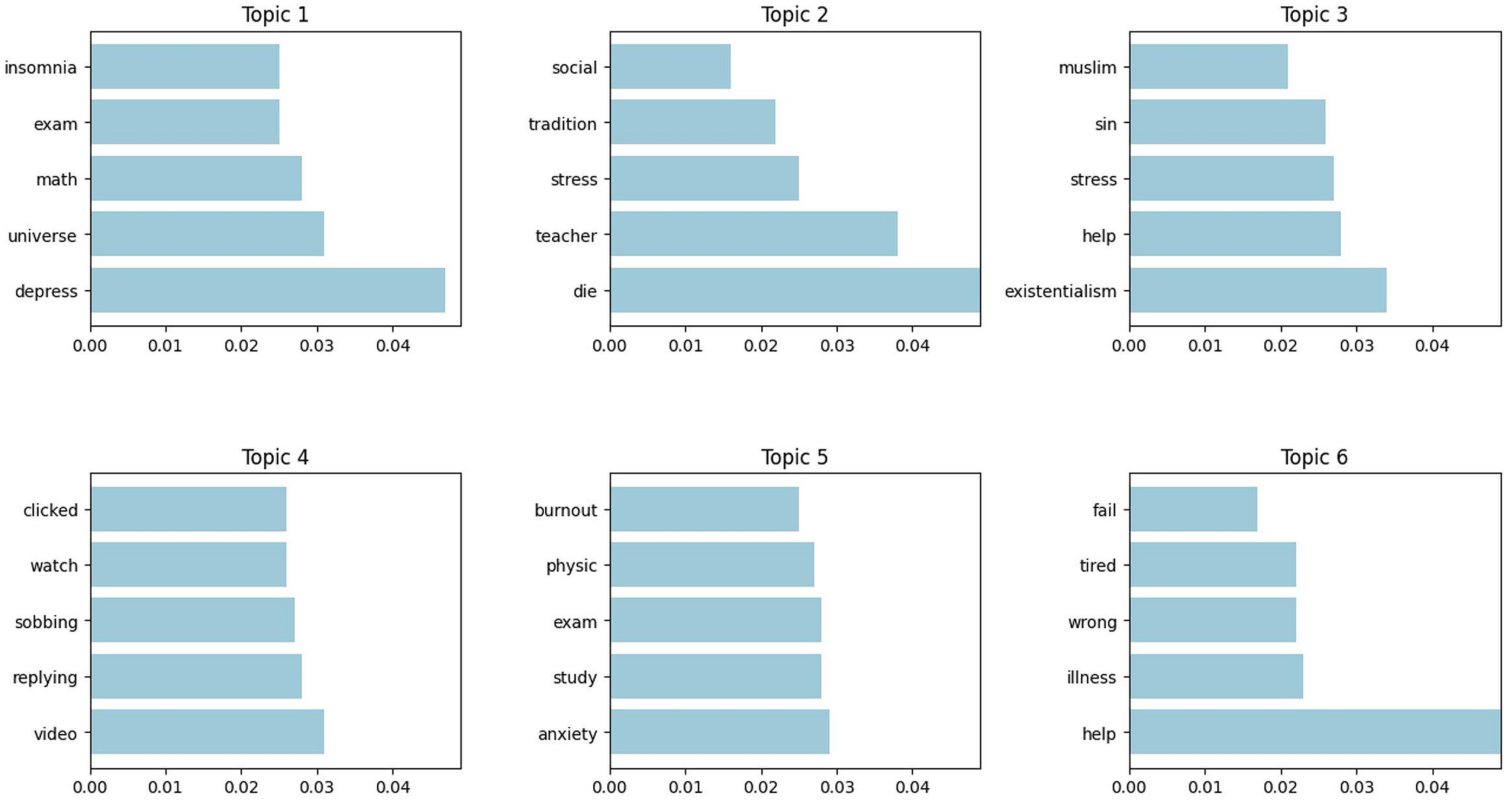
Top 6 topics of LDA.

To further enhance the accuracy of the topic model, particularly when addressing complex semantics and keywords in varying contexts, this study has opted to employ the LDA model for secondary modelling. Through a more nuanced and hierarchical analysis of topic vocabulary, the LDA model proves more effective in uncovering the specific content behind each topic and its underlying context, thus offering a clearer and more precise division of topics for research purposes. As illustrated in Figure 7, the prominent keywords for each topic are presented. Based on the analysis of these keywords, six primary topics were ultimately extracted.

Topic 1: Unexpected Learning Outcomes and Anxiety. The frequent appearance of terms such as “exam” and “math” suggests that academic assessments and mathematics-related subjects may be central sources of academic stress. Moreover, the use of the term “universe” also suggests a possible philosophical introspection among students when faced with scholarly pressures (Bennett et al., 2022; Harrison, 2003), especially regarding the sense of the future and life. This reflective practice could ease some psychosocial loads on students but simultaneously worsen their academic stress. For instance, the incidence of words such as “depress” and “insomnia” indicates the potential link between scholar stress, depression, and sleeping disorders, which in turn further destabilize learning and increase anxiety.

Topic 2: The Interwoven Interaction Between Environment and Stress. Key words such as “teacher,” “social,” and “tradition” explain that the social environment and cultural tradition play a major role towards an individual’s perception of stress. In highly competitive societies particularly, individuals have very many expectations from teachers, families, and society that can heighten their sense of pressure. Under severe stress, individuals can also have destructive attitudes towards life, leading to painful sentiments and exacerbating mental disorders such as depression and anxiety. The employment of words such as “stress” and “die,” for example, conveys a darker and more existential response to the crushing pressure (Ovsiannikova et al., 2024).

Topic 3: The Call for Psychological Assistance and the Conflict of Faith. Existentialism (Macquarrie, 1972) focuses on individual freedom and responsibility, yet also reveals the solitude that human beings feel in the face of uncertainty and tension. The mention of terms like “stress” and “existentialism” suggests that when reacting to life and academic stress, individuals become prone to intense introspection (Sharma and Pestonjee, 2020), challenging philosophical concepts such as the meaning of life and the value of individual existence. At the same time, the use of the words “help,” “sin,” and “Muslim” shows the tendency of the subject to seek outside assistance in times of mental distress. For religious people, psychological problems could be perceived as an indication of “lack of faith.” This creates a dilemma whereby individuals may find it hard to accept modern psychological treatment and support while seeking religious forgiveness and solace (Aguirre Velasco et al., 2020). The psychology versus religion conflict makes the acceptance of professional mental health treatment more complex.

Topic 4: Sentimental Triggers and Psychological Crises under the Platform Effect. The terms “video,” “watch,” and “replying” reflect how video content on social media and online platforms serves as a significant medium for sentimental triggers. The act of clicking on content (“clicked”) reveals how users’ sentimental identification and sentimental responses deepen as they engage with the video material. This effect is particularly pronounced when the content deals with personal struggles, painful experiences, or sentimental conflicts, causing viewers to experience strong sentimental resonance. Specifically, when watching videos that display sentimental expressions, such as “sobbing” viewers’ sentimental fluctuations are heightened, leading to sentimental instability. These sentimental shifts may not only trigger short-term sentimental upheaval but can also escalate an individual’s psychological crisis, potentially exacerbating mental health issues such as anxiety and depression.

Topic 5: Sentimental Anxiety in the Context of Study and Exams. The terms “physics,” “study” and “exam” reflect common sources of stress during the learning and examination process, especially when facing exams in subjects that are more abstract in nature. In science courses (such as “physics”), students often encounter complex concepts that present greater cognitive challenges and uncertainties, which can easily trigger sentimental anxiety. This sentimental anxiety not only intensifies students’ concerns about their academic performance but may also lead to the development of burnout. Burnout causes students to lose interest in their studies, which in turn negatively affects their learning outcomes (Yusof et al., 2023). Words such as “anxiety” and “burnout” are commonly used to describe these sentimental responses.

Topic 6: The Disintegration and Repair of Self-Worth. Use of the terms “wrong” and “fail” indicates how prone people are to doubting their competence in reaction to unfavourable maximum pressures like academic and life challenges. Those feelings of failure tend to reduce confidence levels and dissolve one’s sense of self-worth (Ronnie and Philip, 2021). When these sentiments accumulate over time and reach a critical point, individuals may experience physical exhaustion and health issues (such as “illness” and “tiredness”), which further exacerbates the decline in self-esteem. The interaction between physical discomfort and psychological stress can create a vicious cycle, diminishing the individual’s psychological resilience. In such instances, seeking help from others (as indicated by the word “help”) becomes a crucial avenue for repairing one’s sense of self-worth. By relying on support and encouragement from others, individuals can begin to rebuild their confidence and sense of identity, although this approach may also harbour potential risks.

**Figure 8:**
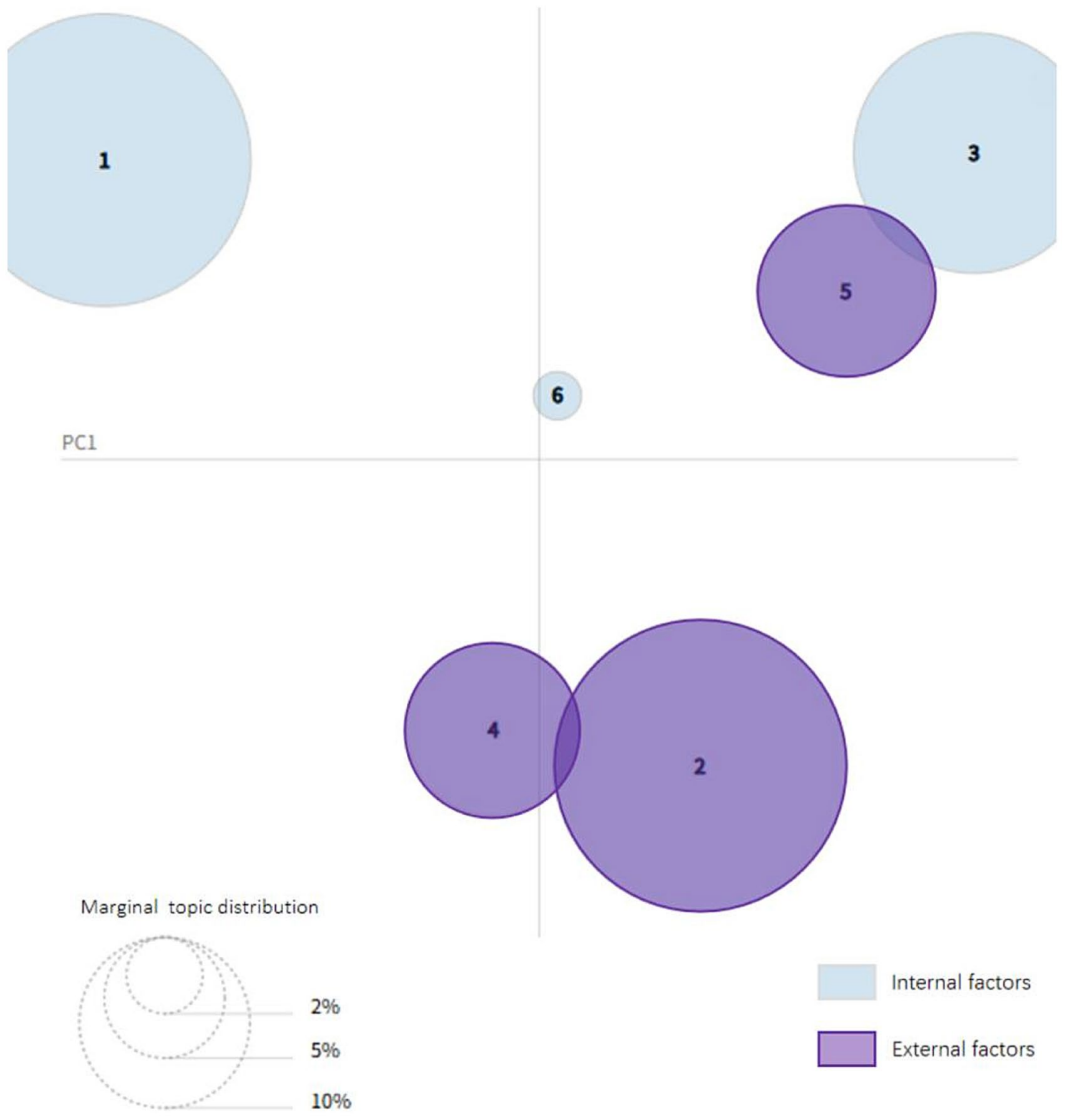
Intertopic distance map.

Based on the above topics, the following conclusions can be drawn: Academic stress is influenced by a variety of factors operating at multiple levels, and a psychological crisis arises when this stress reaches a critical threshold. As illustrated in Figure 8, academic pressure stems not only from internal individual factors, as discussed in Topic 1, Topic 3 and Topic 6, but also from cumulative effects arising from multi-dimensional interactions within the realms of education, society, and culture, as highlighted in Topic 2, Topic 4 and Topic 5. In responding to these pressures, individuals often engage in philosophical reflection to explore the meaning of their own existence, striving to achieve a sense of inner equilibrium. However, the complex interplay of the stressors tends to compound psychological distress, such as anxiety and depression, slowly eroding the psychological resilience of a person. Further, the phenomenon of sentimental contagion of social media and video content can subtly strengthen sentimental oscillations, triggering future psychological crises. In this context, it is crucial that individuals subjected to such colossal stress seek adequate psychological support and assistance to mitigate the ill effect of the pressures.

## 5 Discussion and contribution

Through affect analysis and multi-layered theme modeling, six semantic topics and their sentimental orientations towards academic stress have been identified in this research. Following the coupling process logic of semantic relations between topics and psychological processes, this article introduces the ERA Model as an orderly mechanism to account for the generation of academic stress and the psychological coping processes.

### 5.1 Discussion

#### 5.1.1 Topic discussion

##### 5.1.1.1 Conflict between external expectations and personal abilities

The social environment and cultural traditions play a pivotal role in shaping an individual’s experience of stress (Rusticus et al., 2023). As the primary vessel for institutional pressure, the education system is widely acknowledged as a significant source of academic stress (Högberg, 2024). This is particularly pronounced in the highly competitive arena of higher education, where the pressure to gain admission becomes especially intense (Levine and Van Pelt, 2021). Within such an environment, academic achievement is often reduced to the principal measure of personal success and social identity (Guterman, 2021). Modern educational systems typically emphasise standardised examinations, performance metrics, and ranking competitions, thereby fostering a relentless pressure to achieve high scores and rankings (Ballantine et al., 2021). This, in turn, leads students to internalise academic success as a reflection of their self-worth.

Confronted with the expectations of various stakeholders—society, families, and teachers—students tend to experience heightened psychological pressure due to the high expectations from them, coupled with lower levels of self-efficacy and uncertainty regarding academic outcomes (Fatima et al., 2021; Liem and Senko, 2022; Zhang et al., 2025). This pressure not only constrains students’ sense of value from growing but also causes them to feel that their own identity is dictated by external norms (Katz et al., 2022). For instance, in mathematics classes, struggling students who face challenging ideas and problems will take action initially, only to suffer decreasing results in the long run, which readily degenerates into sentimental anxiety and powerlessness. This, subsequently, can lead to academic burnout, ultimately undermining the entire learning experience (Downing et al., 2020; Musa and Maat, 2021; Suren and Kandemir, 2020).

##### 5.1.1.2 Limitations and breakthroughs of self-regulation ability

During learning, students’ self-regulation ability relies on many unforeseen variables like uncertainty regarding scores in exams, differences in the rate of learning, and time management problems (Hartanto et al., 2021). These problems are dumping too much weight on students’ motivation, sentimental stability, and behaviour control. This is particularly prominent in science and engineering disciplines, where students will overemphasize grades to the neglect of the process of learning the material itself, and therefore adopt a performance strategy to learning (Wang, 2023). This orientation actually raises learning anxiety (Liu et al., 2020). Externally regulated by the standards for motivation, this orientation, in addition to reducing the inner motivation of the students, also renders them more susceptible to the vicious burnout-stress cycle.

Besides, disordered time management also inhibits learning effectiveness more, leading to procrastination and stagnation, while fear of failure makes some students back off or abandon challenges altogether (Wolters and Brady, 2021). All these behaviours point to the lack of resources and regulatory bottlenecks in self-monitoring, sentimental regulation, and the initiation of behaviour, which are indicative of “regulatory ability limitations” as posited under the ERA model. However, empirical evidence suggests that with reconstruction of psychological support and reestablishment of value identity, students are more and more able to overcome these adaptive challenges. Psychological counseling and peer support mechanisms reinforce the self-efficacy of students, thus boosting their sentimental control and coping strategies. Stress belief system is also double-barreled in controlling students’ stress. On the positive side, religion can be a meaningful source to make students better understand themselves and their own worries and needs (Entwistle, 2021). Otherwise, religious and cultural beliefs versus prevailing academic issues today might initiate value conflicts and self-denial (Högberg, 2024; Holmes, 2020; Luna and Draper, 2024). To this end, students will be able to exhibit resilience where they are in the position of being able to formulate responsive mechanisms that are in harmony with their own identity (García-Pérez et al., 2021).

Generally, the dynamics of stress in this model are all those which match well the Regulation mechanism of the ERA model: adaptation of internal resources by the students, sentimental reactions coping, and reaction to outside demands in high-stress environments. This is not merely a psychological regulation challenge, but also an intense process of value integration and responsiveness of the individual.

##### 5.1.1.3 The accumulation and interaction of multiple levels of pressure amplifies academic pressure

As academic stress continues to accumulate at the individual level (Haight et al., 2023), the rise of digital platforms has further transformed the modes through which stress is transmitted and experienced. Many students choose to express or release their academic stress through virtual identities on social media (Lu et al., 2021). Such public or semi-anonymous sentimental narratives can be further amplified when students engage with educational videos or resonant content, creating a collective sentiment contagion effect (Rühlemann, 2022). At the same time, the rapid circulation of social media messages and the density of information can generate sensory overload, thereby expanding both the sources and the frequency of stress signals students are exposed to. This dynamic significantly influences their sentimental states and psychological resilience (James et al., 2022). In the absence of sufficient sentimental support and psychological resources, high-frequency exposure to information within digital environments often intensifies cognitive load and magnifies perceived stress (Feroz et al., 2022). Although sentimental expression on social media may provide a temporary release effect in the short term, the cognitive dissonance between virtual spaces and real-life experiences can aggravate internal psychological conflicts, leading to the worsening of sentimental problems (Appel et al., 2020; Meshi and Ellithorpe, 2021).

Along with this, external stressors—i.e., the educational system, social culture, religious life, and internet sites—when not adequately supported by individual intrinsic factors—e.g., sentimental needs, self-regulatory abilities—are bound to build up dynamically at various levels. Chronic stress is caused by these many-faced stressors, which, over time, strengthen their psychological impacts structurally and in language expression and thus lead to somatic and psychic disorders, like anxiety, depression, and insomnia.

#### 5.1.2 Research limitations

Both the ERA and the Transactional Model of Stress and Coping (Biggs et al., 2017) emphasize the interaction between external sources of stress and individual psychological mechanisms, acknowledging the mediating role of sentiment and cognition in the stress process. However, ERA differs in that it highlights the structural and cultural pressures of educational systems, social media, and other contextual factors in the digital age, proposing that stress is continually amplified in the external environment, leading to more complex cyclical mechanisms. In particular, compared to Self-Determination Theory (Ryan and Deci, 2000), ERA also focuses on motivation and self-regulation. Both frameworks emphasize the tension between external control and intrinsic motivation, but while the core of SDT lies in the continuum of motivation from external control to internalization, ERA places greater emphasis on the interaction between external expectations, regulatory capacity, and amplification effects, incorporating the influence of social structures and media contexts.

**Figure 9:**
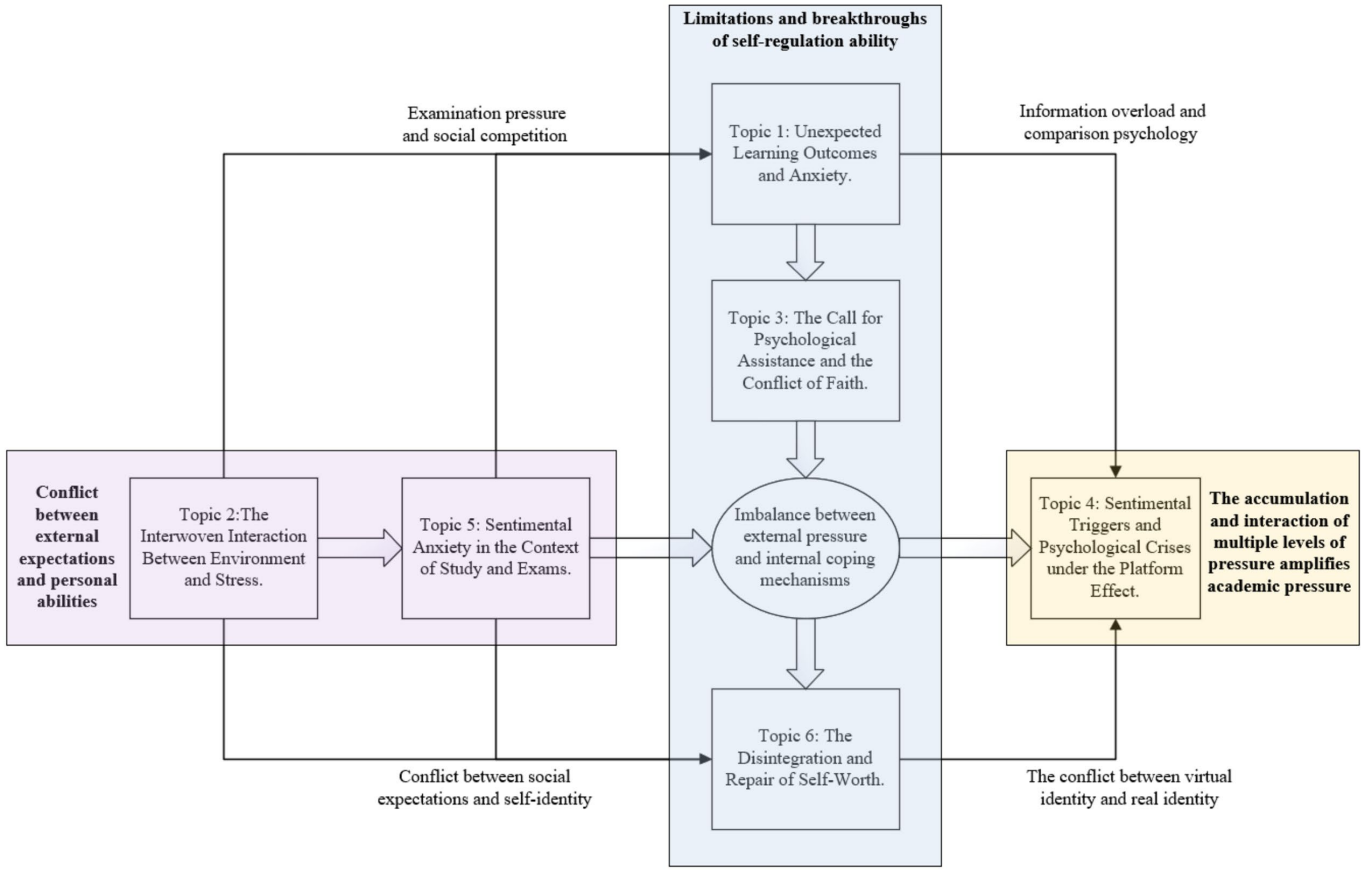
ERA theoretical model.

Thus, ERA builds upon insights from psychological models of motivation and regulation and extends them to the macro-social and cultural levels, offering a more explanatory framework for understanding stress in contemporary educational and societal environments. Figure 9 visually illustrates the core components of the ERA model. Nonetheless, ERA has its limitations. First, its emphasis on external structures and cultural factors may, to some extent, undermine attention to individual agency and differences, marginalizing individual regulation and adaptation strategies in the theoretical explanation. Secondly, ERA’s amplification mechanisms largely remain at the macro and symbolic levels, lacking a systematic explanation of how stress concretely translates into individual experiences and behaviours at the micro-psychological level. Therefore, while ERA expands the perspective of social constructivism, it still needs to integrate with individual-level psychological models to form a more comprehensive theoretical framework.

This study employed BERT sentiment analysis and multi-layer topic modeling to successfully cluster and precisely analyze 33,827 YouTube comments. However, there are some limitations to this research. First, the data was only collected from comments on popular videos from the past 5 years on YouTube, meaning the sampling time and region were relatively limited. Secondly, the current study primarily focuses on the English-language context and does not account for regional differences across various languages and cultural backgrounds. Additionally, the annotation of sentiment labels may be influenced by individual sentimental biases. Future research could validate the model’s universality by using cross-cultural and diverse datasets, optimizing the sentiment analysis model to reduce individual biases and improve accuracy. Therefore, future studies should expand the temporal and spatial scope of data collection, incorporating comments from more platforms (such as Twitter, Instagram, Reddit, etc.) and from different regions and time periods, to build a more diverse dataset for spatiotemporal tracking and other analyses. Moreover, the study should focus on the distinctive characteristics of comments from different languages, cultures, and communities, further enhancing the universal applicability and cross-cultural adaptability of sentiment analysis models. Additionally, the sentiment analysis model should be optimized by introducing more contextual information and finer-grained sentiment analysis layers, reducing individual sentimental biases, and improving the objectivity and accuracy of sentiment labels.

### 5.2 Research contributions and inspiration

#### 5.2.1 Research contributions

##### 5.2.1.1 Blend of methods and synergistic strengths of BERTopic and LDA

The paper presents a novel combination of BERTopic and the Latent Dirichlet Allocation (LDA) model, leveraging the capabilities of the two for thematic modeling tasks through their functional complementarity. Although both possess strength and weakness, BERTopic’s strength in processing huge, unstructured corpora, complemented by LDA’s strengths in topic smoothing, coherence, and semantic precision, forms a robust text analysis tool with both macro- identification and micro-interpretation capabilities. This integrative approach not only enhances implicit sentimental cueing and polysemy identification but also enlarges the methodological boundaries of thematic modeling and sentiment analysis at the corpus semantic level, with new technical methods and empirical evidence to analyze complex psychological language data.

##### 5.2.1.2 Construction and application of the ERA theoretical model

The ERA theoretical model proposed in this study is a unified and dynamic frame of reference for explaining academic stress generation mechanisms and psychological regulation processes. The model, based on six basic semantic themes, dissects the inherent psychological mechanisms in place and exposes the external expectation, self-regulation resource, and structural amplification effect interplay driving academic pressure. The ERA framework not only describes how stress builds up, is converted, and regenerates, but also underscores the shaping influence of the digital world on mental dilemmas. Indeed, symbolic pressures inherent to language, as they are actualized in social communication, media communication, and algorithmic worlds, are able to heighten individuals’ levels of anxiety and cognitive bias (Walther and Whitty, 2021; Zhang et al., 2021). In this regard, the theoretical framework developed within this study presents a useful theoretical material and basis for further research on how digital narratives create psychological pressure, and how language used on social media structures sentimental construction and psychological intervention.

#### 5.2.2 Research enlightenment

##### 5.2.2.1 Developing a robust sentimental support system

This recommendation directly addresses the conflict between external expectations and self-capabilities and highlights that when students face multiple expectations from family, society, and academics, they often experience negative sentiments such as anxiety, stress, and disappointment, which exacerbate academic pressure. To help students effectively cope with this challenge, it is recommended to introduce long-term mental health support programs, particularly peer support networks (Shalaby and Agyapong, 2020), to provide sentimental support when students face academic stress and reduce their excessive sensitivity to external expectations (Weisberg et al., 2023). Additionally, schools should work closely with teachers and parents (Li, 2022) to identify students who are experiencing significant academic pressure and provide timely interventions (Crothers et al., 2020), ensuring that students can maintain their mental health while coping with external pressures.

##### 5.2.2.2 Emphasizing individualized learning strategies and belief value modification

This recommendation directly addresses the issue of the limitations and breakthroughs of self-regulation ability, noting that students’ capacity for self-regulation is influenced by various unpredictable factors and is often challenged when confronted with academic stress. There are numerous factors beyond control that influence students’ self-regulation ability, and these need to have learning strategies adapted to particular conditions in an effort to build psychological resilience (Barbosa et al., 2024; Shemshack and Spector, 2020). For students with religious beliefs, schools can offer cultural adaptation and value alignment education, assisting students in reconciling any potential conflicts between their faith and academic pursuits (Poitras Pratt and Gladue, 2022).

##### 5.2.2.3 Prioritising the regulation mechanisms of information overload on digital platforms

This recommendation directly addresses the issue of the accumulation and interaction of multiple levels of pressure amplifying academic stress, highlighting that when students face multiple sources of pressure—particularly high-density online information flow—it leads to anxiety, distracted attention, and ultimately intensifies academic pressure. To mitigate the risks associated with prolonged exposure to dense information flows, both schools and families should collaboratively provide time management tools, aiding students in effectively organising their online time (Yeung and Yau, 2022). Based on this, the platforms can implement intelligent language recommendation mechanisms and personalized information provision channels with particular emphasis on underage users. Strictly controlled mechanisms can be initiated to limit the spread of irrelevant or harmful content (Pan, 2023) so as to effectively counter the harmful impact of information overload on psychological health and academic stress.

## 6 Conclusion

This study employed topic and sentiment analysis of academic stress discourse using the BERTopic and LDA models, and disclosed multiple sources of academic stress and its semantic build-up in an online setting. The findings show that the breakdown of external expectations and internal capabilities constitutes a main driver of academic stress, particularly in the framework of a competitive academic environment. The mismatch between the high standards of society, families, and schools and the students’ sense of self-efficacy often leads to heightened sentimental distress and psychological tension. Furthermore, without resources and self-regulation skills—time management, motivation, and sentimental responding capability—stress accumulates, exacerbating the situation. The cultural adaptability and presence of psychological support could be the solution in curbing these limitations. In addition, the external pressure from the online platforms, integrated with the total social and cultural structure, amplifies stress perception through an interactive process of amplification culminating in a dynamic interaction of a number of stress factors. These results lay the foundation for building the ERA Model, where authors suggest a three-phase dynamic process: from external expectation to internal regulation, and eventually to structural effect.

Although this study provides an in-depth analysis of the multiple sources of academic stress and their cumulative effects through multi-modeling, there are still limitations in its process. Future research could integrate richer data sources and enhance accuracy. As a direction for development, BERTopic (Raman et al., 2024) and the LDA model (Choubey et al., 2020) could be used for in-depth measurement and analysis of data such as news reports, forum discussions, and interdisciplinary academic literature. At the same time, educators and psychological counselors should, based on the research findings, offer more refined and personalized intervention strategies that consider individual differences, thus better assisting students in coping with academic stress and fostering the development of their psychological resilience and well-being. This approach not only addresses sentiments related to academic pressure but also ensures that interventions are aligned with the sentimental and psychological needs of students.

## Data Availability

The raw data supporting the conclusions of this article will be made available by the authors, without undue reservation.
